# Caring Self-Efficacy of Personal Care Attendants From English-Speaking and Non-English-Speaking Countries Working in Australian Residential Aged Care Settings

**DOI:** 10.1177/08982643231183466

**Published:** 2023-06-14

**Authors:** Sumina Shrestha, Yvonne Wells, Christine While, Muhammad Aziz Rahman

**Affiliations:** 1 Australian Institute for Primary Care & Ageing, 2080 La Trobe University, Melbourne, VIC, Australia; 2 Institute of Health and Wellbeing, 509986 Federation University, Berwick, VIC, Australia; 3 Department of Non-Communicable Diseases, Bangladesh University of Health Sciences (BUHS), Dhaka, Bangladesh; 4 Faculty of Public Health, Universitas Airlangga, Surabaya, Indonesia

**Keywords:** Australia, caring, confidence, migrants, personal care attendants, nursing homes, self-efficacy

## Abstract

**Objectives:** This study compared the caring self-efficacy between personal care attendants (PCAs) from English-speaking and non-English-speaking countries, controlling for potential sociodemographic and work-related covariates. PCAs’ perceptions of their caring self-efficacy were further explored. **Methods:** An independent samples *t*-test was used to determine the mean difference in the caring self-efficacy score between the two groups. A multivariate analysis was conducted to adjust for covariates. Thematic analysis was conducted on open-ended responses. **Results:** The results showed that caring self-efficacy was significantly influenced by whether participants primarily spoke English at home rather than where they were born. Younger age and everyday discrimination experiences were negatively associated with caring self-efficacy. Both groups perceived that inadequate resources and experiencing bullying and discrimination reduced their caring self-efficacy. **Discussion:** Access to organisational resources and training opportunities and addressing workplace bullying and discrimination against PCAs, particularly younger PCAs and those from non-English-speaking backgrounds, could improve their caring self-efficacy.

## Background

In recent years, residential aged care settings have become more culturally and linguistically diverse than ever in member countries of the Organisation for Economic Co-operation and Development (OECD), including Australia (Australian Government Department of Health, 2020; Colombo et al., 2011). In Australia, as of June 2020, 19.7% of the older adults using permanent residential care services were from non-English-speaking backgrounds (NESB), and 9.3% preferred a language other than English to communicate (Australian Institute of Health and Welfare, 2021).

The direct care workforce in Australian residential aged care facilities (RACFs) is even more culturally diverse than the residents, with 35% born in NESB countries (Australian Government Department of Health, 2020). Of these NESB direct care staff, 72% work as personal care attendants (PCAs), the primary staff group providing direct care to residents. PCAs are often referred to as personal care workers, nursing aides, nursing assistants, or care aides. The proportion of PCAs employed in residential aged care born in NESB countries is 36% (Australian Government Department of Health, 2020). Recently, the Australian government has been considering using immigration to compensate for the growing aged care labour shortage (Morrison-Dayan, 2019; Thompson, 2022), which may have the impact of further increasing the proportion of PCAs from NESB countries.

Caring for older people who cannot live independently without assistance is a challenging occupation comprising comprehensive physical, emotional, social, spiritual, and environmental care (Australian Productivity Commission, 2011). Caring self-efficacy in aged care refers to the perceived ability of care workers to deal appropriately during challenging situations, accept constructive criticism regarding their work, be compassionate towards older residents, and be aware of their actions as care workers, as well as recognise the positive impact of their work (Shrestha et al., 2021). The demanding, complex, and multifaceted nature of care may influence the caring self-efficacy of aged care workers. Studies have found that care workers who communicate well are more confident, fostering interpersonal relationships with care recipients and coping better with a broad range of stressors in their workplace (Leal-Costa et al., 2020). Having a shared culture and language facilitates aged care workers’ communication with older residents, boosting their self-confidence to do their job (Strandroos & Antelius, 2017).

Studies have shown that PCAs from NESB countries are more underprivileged in the aged care labour market than their co-workers from English-speaking backgrounds (ESBs) (Charlesworth & Isherwood, 2021). They are mainly employed as casual staff, often without employment benefits, and are offered shorter shifts, more irregular hours, lower pay, and more challenging residents to care for than their ESB counterparts (Charlesworth & Isherwood, 2021; Colombo et al., 2011). They may also experience racial discrimination from residents and co-workers (Stevens et al., 2012). Evidently, limited English language proficiency is the most common challenge for PCAs from NESB countries (Chen et al., 2020; Gao et al., 2015).

Despite the significant representation of PCAs from NESB countries in residential aged care, there is still a dearth of literature exploring their self-efficacy in caring for older residents. It is also possible that the confidence level of English-speaking PCAs may be negatively affected by the increasing proportion of residents and co-workers from many different cultural and linguistic backgrounds. Moreover, the impact of the recent COVID-19 pandemic on the well-being of residential aged care staff has been unprecedented, with increased work pressure, higher frustrations, and feeling undervalued (Brydon et al., 2022; Krzyzaniak et al., 2021). Hence, this study aimed to compare the caring self-efficacy of PCAs born in English-speaking and non-English-speaking countries while adjusting for potential covariates. We hypothesised that PCAs born in English-speaking countries would have higher caring self-efficacy than those born in non-English-speaking countries. In addition, this study explored PCAs’ opinions on what may have affected their caring self-efficacy using open-ended question.

The analysis of caring self-efficacy was adjusted by the inclusion of sociodemographic and work-related variables and PCAs’ everyday discrimination experiences to address their potential confounding effect on the relationship between caring self-efficacy and the cultural/linguistic backgrounds of PCAs. Sociodemographic variables included Age, Gender, Marital status, Education, Country of birth, Primary language spoken at home, and Extent to which participants felt Australian, while work-related variables included Informal care experience, Years of experience, Employment status, Working hours, Job satisfaction, Quality of initial training, Type of facility, Intention to remain in the aged care sector, and Perceived influence of COVID-19. These variables were included as control variables because previous evidence had shown that PCAs from NESB countries and their peers from ESB countries differ on most sociodemographic and work-related indicators (Charlesworth & Isherwood, 2021; Spencer et al., 2010). NESB PCAs face discrimination based on their language, race, accents, and skin colour in RACFs (Nichols et al., 2015; Walsh & Shutes, 2013). In addition, many of these variables are related to the main outcome. A scoping review by Shrestha et al. (2021) showed that ethnicity, age, and job-related factors (such as job satisfaction or work experience) were likely to influence the caring self-efficacy of PCAs.

## Materials and Methods

### Study Design and Setting

A cross-sectional, embedded mixed-methods study was carried out in residential aged care facilities in Australia in 2021–22. This study was a predominantly quantitative study with a supplementary qualitative component.

### Study Population and Inclusion Criteria

The study population comprised PCAs working in residential aged care settings across Australia. PCAs were eligible to participate if they (a) had a certificate III or IV training in individual support (ageing), a minimum requirement to work as a PCA in residential aged care in Australia, and (b) had been providing care to older residents in residential aged care for at least a month.

Study participants were grouped by whether they were born in an ESB or NESB country. An intermediate level of English language is expected in Australia to work as a PCA in RACFs.

### Sample Size

A minimum sample size of 128 in the ESB group and 64 in NESB was determined as adequate to test the statistical difference between two independent means with power (1 − *β*) of 90%, significance criterion (*α*) of .05, medium effect size (ES) of .50 (Cohen, 1988), and allocation ratio (*N*1/*N*2) of 2. The required sample size was calculated using G-power software (Faul et al., 2009). The final analysis included 192 participants from English-speaking countries and 88 from non-English-speaking countries.

### Participant Recruitment and Data Collection

An online survey link was developed to collect the data. Convenience sampling was used to recruit study participants. Twenty-five randomly selected residential aged care facilities listed by the Australian Government (Australian Institute of Health and Welfare, 2019) were first approached to recruit participants. However, due to increasing COVID-19 cases in the facilities during the data collection phase, only two facilities forwarded our survey link to their eligible staff. Because of the low response rate from facilities, online platforms such as aged care workers’/PCAs’ forums on social media and emails to relevant organisations (such as HelloCare Australia) were utilised to disseminate the survey link to recruit enough participants for the study. Data were collected between August 2020 and March 2021.

The online survey included two components: (a) a participant information statement explaining research aims, potential benefits and risks of participation, and the sources of support and (b) the questionnaire. Participants had the opportunity to read the participant information statement and choose whether to participate or withdraw from the survey. They could also stop or withdraw participation before submitting their responses. The questionnaire was completely anonymous to protect the privacy of the participants.

Ethical approval for the study and required amendments to the procedure were received from the Human Ethics Committee of La Trobe University, Australia (HEC20039).

### Measures

A self-administered structured questionnaire comprising questions on sociodemographic characteristics, everyday discrimination, work-related variables, and caring self-efficacy was used to collect the data.

#### Sociodemographic Variables

Variables related to sociodemographic characteristics included Age, Gender, Marital status, Education, Country of birth, Primary language spoken at home, Extent to which participants felt Australian, and Everyday discrimination experience (see Table 1 for response options).

**Table 1.** A Comparison of Sociodemographic and Work-Related Characteristics Between English Speaking and Non-English Speaking Nursing Assistants.

Variables	Categories	English Speaking		Non-English Speaking		*p*-Value
		*n*	%	*n*	%	
Age	Mean ± *SD*	43.3 ± 11.9		32.7 ± 9.2		<.001^*^
Gender	Male	3	1.6	12	13.6	<.001^*^
	Female	189	98.4	76	86.4	
Marital status	Never married	75	39.1	19	21.6	.004^*^
	Ever married	117	60.9	69	78.4	
Feeling Australian	Very/somewhat Australian	189	98.4	52	59.1	<.001^*^
	Not very/not at all Australian	3	1.6	36	40.9	
Language spoken at home	English	189	98.4	25	28.4	<.001^*^
	Other language	3	1.6	63	71.6	
Educational level	Certificate III or IV	144	75.0	20	22.7	<.001^*^
	Diploma or degree	48	25.0	68	77.3	
Everyday discrimination	Mean ± *SD*	25.1 ± 9.4		21.5 ± 8.2		.003^*^
Informal care experience	Yes	140	72.9	67	76.1	.569
	No	52	27.1	21	23.9	
Years of experience	Mean ± *SD*	9.3 ± 8.4		4.3 ± 5.0		<.001^*^
Intention to remain in aged care job	Yes	144	75.0	48	54.5	.001^*^
	No	48	25.0	40	45.5	
Current employment status	Permanent	154	80.2	65	73.9	.232
	Casual/contract	38	19.8	23	26.1	
Working hours	Full-time	46	24.0	17	19.3	.380
	Part-time	146	76.0	71	80.7	
Type of facility	Private	86	44.8	47	53.4	.180
	Others	106	55.2	41	46.6	
Job satisfaction	Satisfied	138	71.9	81	92.0	<.001^*^
	Dissatisfied	54	28.1	7	8.0	
Quality of training	Excellent/good	155	80.7	82	93.2	.007^*^
	Fair/poor	37	19.3	6	6.8	
Influence of COVID pandemic on the CES	Yes	22	11.5	7	8.0	.372
	No	170	88.5	81	92.0	

*p-value < .05.

The perceived level of discrimination experienced by study participants was measured using an Everyday Discrimination Scale. This scale includes nine items, with response options along a 6-point Likert-type scale, ranging from 1 (Strongly disagree) to 6 (Strongly agree). This scale has been validated and used among the general population, including Indigenous people and immigrants, in community settings, healthcare settings, and other workplaces (Agrawal et al., 2018; Kim et al., 2014; Thurber et al., 2021). The internal reliability of Everyday Discrimination Scale in our study was good – Cronbach’s alpha value of .87.

#### Work-Related Variables

Work-related variables comprised whether respondents had Informal care experience, Years of experience, Employment status, Working hours, and Type of facilities they worked in. Participants were also asked to rate the Quality of their initial training, Job satisfaction, Intention to remain in the aged care sector, and Perceived influence of the COVID-19 pandemic on their caring self-efficacy (see Table 1 for response options).

#### Caring Efficacy Scale

Questions on caring self-efficacy were based on the ‘Caring Efficacy Scale’. Coates (1997) recommended the original 30-item scale to assess nurses’ and other caregivers’ beliefs about their caring self-efficacy. Reid et al. (2015) assessed the scale’s psychometric properties among Australian nurses working in the health care sector, including aged care, and recommended a 28-item scale comprising two sub-scales: *‘Confidence to Care’* and *‘Doubts and Concerns’*. However, the psychometric properties of this scale had not previously been tested among PCAs in aged care.

Therefore, before comparing the caring self-efficacy between ESB and NESB participants, we conducted a confirmatory factor analysis of the 30-item one-factor model (Coates, 1997) and the 28-item two-factor model (Reid et al., 2015). However, both models had a poor fit in our study population. Therefore, exploratory factor analysis was conducted to examine the dimensionality and underlying structure of the Caring Efficacy Scale. This analysis resulted in two factors, and 22 items out of 30 were retained (see Table 2 for the final scale). This 22-item scale showed satisfactory internal consistency – Cronbach’s alpha coefficient for the scale was .85 (Shrestha et al., 2023). Hence, the modified 22-item scale was used to assess the caring self-efficacy of study participants in the final analysis.

**Table 2.** Caring Efficacy Scale With 22 Items.

Factor 1: Confidence to Care
I can usually create some way to relate to almost any resident
I can usually get residents to like me
I am able to tune into a particular resident and forget my personal concerns
Residents can tell me almost anything and I won’t be shocked
It is easy for me to consider the multi-facets of a resident’s care, at the same time as I am listening to them
I can walk into a room with the presence of serenity and energy that makes residents feel better
I use what I learn in conversations with residents or their family to provide more individualised care
I have the ability to introduce a sense of normalcy in stressful conditions
I can usually establish a close relationship with residents
I convey a sense of personal strength to residents in the course of caregiving
Factor 2: doubts and concerns
I lack confidence in my ability to talk to residents from cultural & linguistic backgrounds different from my own
I often find it hard to relate to residents from a culture different than mine
I often become overwhelmed by the nature of the problems residents are experiencing
Even when I am feeling self-confident about most things, I still seem to be unable to relate to residents
When trying to resolve conflict with a resident, I usually make it worse
I often find it hard to get my point of view across to residents when I need to
I feel if I talk to residents on an individual, personal basis, things might get out of control
I don’t feel strong enough to listen to the fears and concerns of my clients
I have difficulty suspending my personal beliefs and biases in order to hear and accept a resident as a person
I seem to have trouble relating to residents
If I find it hard to relate to a resident, I will stop trying to work with that person
Even when I really try, I can’t get through to difficult residents

Likert-type scale values of items in the modified scale were converted from ‘−3 to +3’ to ‘1 to 6’, with response options ranging from strongly disagree to strongly agree. Items in the *Doubts and Concerns* subscale were reverse-scored, so six represented the highest self-efficacy for each item. Hence, *Doubts and Concerns* refers to *(No) Doubts and Concerns.*

### Data Analysis

Quantitative analysis was conducted to compare the caring self-efficacy scores of PCAs from ESB and NESB countries and adjust the influence of country of birth with potential sociodemographic and work-related covariates to identify whether PCAs’ country of birth independently influences their caring self-efficacy. Qualitative analysis was carried out to explore other factors influencing caring self-efficacy from the perspective of the PCAs.

#### Quantitative Data Analysis

Quantitative data were analysed using IBM SPSS version 27.0. Records with incomplete or irrelevant information were excluded during data cleaning.

Descriptive statistics such as means and percentages were calculated for sociodemographic and work-related variables. The total scores on the Everyday Discrimination Scale and Caring Efficacy Scale and subscales for each participant were calculated by summing item scores. The two groups were compared on sociodemographic and work-related characteristics using *t*-tests for continuous variables and the *χ*^2^-test for categorical variables.

Similarly, the difference in mean caring self-efficacy scores between ESB and NESB groups was tested using an independent samples *t*-test. A Welch’s *t*-test was chosen because of unequal sample sizes and variances between these two groups. We then conducted multivariate modelling using a purposeful selection of variables following Bursac’s regression method (Bursac et al., 2008) to examine sociodemographic and work-related covariates that could be associated with caring self-efficacy. For this, simple linear regression was carried out first to check the bivariate association of covariates with caring self-efficacy. In the second step, all the variables with a *p-*value <.25 in the bivariate analysis were included in a multivariate analysis. In the third step, covariates with a *p*-value of less than .05 in step 2 were entered into multiple regression analysis. A model including only significant variables at *p-*value <.05 was identified. The model was then run with variables excluded in step 3 and step 2, adding one variable at a time to the model to see whether variables were statistically significant or whether any variables altered the coefficients of other variables in the model by more than 20%. Any variable that altered coefficients in the model by more than 20% was considered a confounder and was retained in the model.

Model diagnostics were run to check for multivariate normality, multicollinearity, and heteroskedasticity. We observed heteroskedasticity in the multivariate model, but the log transformation did not improve the model. Therefore, heteroskedasticity was addressed using Estimates with Robust Standard Errors (Field, 2017). The effect size of the final model on caring self-efficacy was also calculated.

The same analysis was applied to the subscales ‘*Confidence to Care’* and ‘*(No) Doubts and Concerns’*.

#### Qualitative Data Analysis

Participants were given an opportunity to explain what could have influenced their responses to any of the previous questions related to their experiences as a PCA. Similarly, for participants who said yes to the influence of the COVID-19 pandemic in a structured question, an open-ended question on how the pandemic affected their responses was asked. Qualitative data were analysed in NVivo version 12.0. An inductive approach to thematic analysis was carried out to analyse data generated from the open-ended question. Firstly, the first author read the responses multiple times. Then, an initial list of codes was created, where each code included responses with similar meanings. In the second stage, the first and third authors were involved in grouping codes under potential themes. Finally, the first three authors reviewed and discussed potential themes and agreed on the final themes.

Fifty codes were identified and grouped under five main themes. The rigour of the findings was enhanced by identifying representative quotes (by consensus of all authors) to ensure that the findings were consistent with the participants’ descriptions.

## Results

A total of 280 participants were included in the final quantitative analysis. Initially, 399 potential participants began the survey, but 108 (27.1%) did not progress further. Six potential participants did not complete the caring efficacy scale, while five submitted irrelevant data with the same response option to all statements in the scale.

### Sample Characteristics

The mean age of study participants included in the analysis was 40.0 (±12.2) years. Nearly 95% of the participants were women. Four-in-10 participants had higher educational qualifications than the minimum required training to work as a PCA in Australia. Nearly 64% of participants were born in Australia, while 5% were born in other English-speaking countries (United Kingdom, Ireland, and New Zealand). Among PCAs born in ESB countries, four had parents who were both born in NESB countries, but only one participant had parents who had both migrated from a non-European country. Around 31% were from non-English speaking countries, of which nearly 80% were from Asian countries, mainly from Nepal. Most NESB participants spoke Nepali, and a few spoke Hindi, Filipino, Malayalam, Bahasa, Telugu, Tamil, Afrikaans, Spanish, or Dzongkha. Only 24% of the participants used languages other than English at home.

#### Sociodemographic and Work-Related Characteristics of English and Non-English-Speaking PCAs

Table 1 shows that PCAs from ESB countries were significantly older than those from NESB countries (mean difference: 10.6 ± 1.3, *p* < .001). Male PCAs comprised a higher proportion of respondents among participants from NESB countries than in the ESB sample. PCAs from NESBs were also more likely to have ever been married and to have higher academic qualifications than those from ESB countries. When asked how Australian they felt, those from NESB countries felt less Australian than those from ESB countries. Unexpectedly, the ESB group reported a significantly higher incidence of everyday discrimination than the NESB group.

Regarding work-related characteristics, participants from ESB countries had more work experience and were more likely to continue working in aged care in the future than their counterparts. In contrast, they were more likely to rate their Certificate III/IV training as unsatisfactory and were less satisfied with their job than the NESB group. There were no differences in informal care experience, employment status, working hours, type of facility they were working in, or the influence of the COVID-19 pandemic on their confidence to care.

### Caring Self-Efficacy

The mean score of caring self-efficacy of the study participants was 115.1 ± 12.5 (out of 132), indicating high caring self-efficacy. The mean scores of subscales *Confidence to Care* and *(No) Doubts and Concerns* were 54.7 ± 5.8 (out of 60) and 60.3 ± 8.7 (out of 72), respectively. Descriptive findings related to the Caring Efficacy Scale and subscales are provided in the supplementary file.

#### Differences in the Caring Self-Efficacy Between PCAs from English-Speaking and Non-English-Speaking Countries

The mean scores of the Caring Efficacy Scale and subscales, Confidence to Care and (No) Doubts and Concerns, of participants from ESB countries were significantly higher than those from NESB countries (Table 3). The mean caring self-efficacy score of participants from ESB countries was 117.4 ± 11.1, while it was 109.8 ± 13.8 for the NESB group.

**Table 3.** Comparison of Caring Self-Efficacy Between English Speaking and Non-English Speaking Nursing Assistants.

Variable	Mean Caring Self-Efficacy Score (SD)		Mean Difference	*p*-Value	Cohen’s d
	English Speaking	Non-English Speaking			
Caring self-efficacy	117.4 ± 11.1	109.8 ± 13.8	7.6	<.001^*^	.6 (.4–.9)
Confidence to care	55.9 ± 4.8	52.2 ± 6.8	3.7	<.001^*^	.7 (.4–.9)
(No) doubts and concerns	61.6 ± 8.0	57.7 ± 9.6	3.9	.001^*^	.5 (.2–.7)

*p-value < .05.

#### Multivariate Modelling of Caring Self-Efficacy With Potential Sociodemographic and Work-Related Covariates

Consistent with the analytic plan, Country of birth (ESB vs. NESB), Age, Feeling Australian, Language spoken at home, Education, Everyday discrimination experience, Intention to stay in the aged care sector in the future, Years of experience, Employment status, and Working hours (all of which were significant bivariate predictors of Caring self-efficacy; see Table 4) were included in the initial multivariate model. Only Language spoken at home and Everyday discrimination were independent predictors of Caring self-efficacy. Age was significantly associated with Caring self-efficacy in the fourth step of the regression analysis. None of the covariates was identified as a confounder. Variables in the final regression equation were Age, Primary language spoken at home, and Everyday discrimination. In summary, we found that PCAs who were older, used English as a primary language at home, and experienced less discrimination in their daily life had higher caring self-efficacy than those who were younger, spoke a language other than English at home, and experienced more discrimination (Table 5).

**Table 4.** Bivariate Association of Covariates With Caring Self-Efficacy.

Variables	Unstandardised Coefficients B (95% CI)	Standard Error SE	Standardised Coefficients *β*	*p-*Value
Country of birth (English vs. non-English)	7.6 (4.6–10.7)	1.6	.3	<.001*
Age	.3 (.2–.4)	.1	.3	<.001*
Gender	.3 (−6.3–6.8)	3.3	.005	.938
Marital status	−.5 (−2.6–3.7)	1.6	−.02	.742
Feeling Australian	6.4 (2.2–10.6)	2.1	.2	.003*
Language spoken at home	9.7 (6.4–13.0)	1.7	.3	<.001*
Educational level	−4.8 (−7.8 – (−1.9))	1.5	.2	.001*
Informal care experience	.2 (−3.1–3.6)	1.7	.008	.893
Intention to remain in aged care job	3.7 (.6–6.9)	1.6	.1	.021*
Years of experience as NA	.3 (.1–.5)	.1	.2	.002*
Employment status	2.7 (−.9–6.2)	1.8	.1	.143#
Working hours	3.8 (.3–7.3)	1.8	.1	.034*
Facility type	−.1 (−3.1–2.9)	1.5	−.004	.953
Job satisfaction	2.0 (−1.6–5.6)	1.8	.1	.274
Quality of training	1.8 (−2.3–5.9)	2.1	.1	.384
Everyday discrimination scale	−.2 (−.4 – (−.1))	.1	−.2	.004*
Covid	−1.7 (−6.6–3.1)	2.5	−.04	.489

*p-value < .05; ^#^*p*-value < .25.

**Table 5.** Final Multivariate Model of Factors Predicting Caring Self-Efficacy of Nursing Assistants With Heteroskedasticity-Consistent Standard Errors.

Variables	Unstandardised Coefficients B (95% CI)	Robust Standard Error SE	Standardised Coefficients *β*	*p*-Value
Language spoken at home	8.9 (4.9–12.8)	2.0	.316	<.001*
Everyday discrimination experience	−.3 (−.5–(−.1))	0.1	−.193	.001*
Age	.2 (.03–.3)	0.1	.163	.020*

*F* (3, 276) = 20.4, *R*^2^ = 18.2.

*p-value < .05.

The identified set of predictors accounted for approximately 18.2% of the variation in the caring self-efficacy of PCAs. The joint effect size of the predictors on caring self-efficacy was .22, indicating a medium effect size.

We further conducted multivariate modelling for each subscale separately. Along with speaking English at home and having low everyday discrimination experience, having a full-time position was also significantly positively associated with *Confidence to Care. (No) Doubts and Concerns* were significantly higher among PCAs who were older, spoke English at home, experienced low everyday discrimination, and had higher job satisfaction.

### Qualitative Findings on Factors Influencing Caring Self-Efficacy of PCAs

Participants were asked if they would like to explain what could have influenced their responses to caring self-efficacy. Forty-three participants responded to the question. Five main themes emerged from the thematic analysis of the responses.

#### Organisational Barriers

The organisational barriers theme was divided into two sub-themes:

##### Lack of Resources

Shortages of staff and time were the most discussed issues related to caring self-efficacy. Participants from both ESB and NESB countries reported that a low staffing ratio did not allow them to provide relational and emotional aspects of care, although they believed that they could provide excellent care to the older residents if they had enough time and resources:

*“I feel like I can provide excellent care, and [I] am able to build better relationships with residents if aged care facilities gave us staff more time to do so. We don’t get enough time to do the bare necessities, such as making people clean and feeding them and changing their clothes, let alone have a conversation with them or spend time doing activities with them.” [Participant from ESB country]*

*“Maybe working almost every day with short staff and [we’ve] got to finish our job” [Participant from NESB country]*

##### Support and Recognition

Aged care was viewed as a difficult job. A participant from an ESB country discussed the lack of training provided to assist staff to manage complex care needs as a reason why she found the job difficult:

*“Aged care is a very difficult job, and with little to no support, [having] little to no training in certain areas such as mental health, dementia, and complex behaviours makes it very difficult!” [Participant from ESB country]*

PCAs from NESB countries reported that managers or supervisors were often not reachable, and their requests were not heard, making it difficult to perform their job effectively:

*“[The] manager or care managers never seem reachable when on call. RNs have no clue about [the] residents and treat [PCAs] with no respect, even though they always say [we] work as a team.” [Participant from NESB country]*

*“I am looking after a Greek resident. She [can] only speak and understand Greek, but I don’t. I requested [a change] to my supervisor, but no progress.” [Participant from NESB country]*

#### Challenging Behaviours of Residents

The challenging behaviours of residents were discussed by PCAs from ESB countries only. Several participants reported that such residents’ behaviours were mostly because of their illnesses, making their work difficult.

*“We get hit, kicked, bitten, urinated on, vomited on, defecated on and spat at, as well as sworn at on a regular basis. Many are not able to control their actions, but there are a few (these are generally very much a minority) that make it their mission to make our lives as miserable as their own.” [Participant from ESB country]*

Some participants reported that they should be able to exercise their own judgement about when to persist and when to retreat in those situations:

*“You have to know when to walk away from difficult situations” [Participant from ESB country]*

#### Bullying and Discrimination in the Workplace

Bullying and discrimination by supervisors, colleagues, and residents were also reported to affect participants’ caring self-efficacy. While one ESB participant reported bullying from older staff who had been working longer in aged care, migration status and racial backgrounds were the main reasons for discrimination against NESB participants:

*“Working with a large group of women when you are younger, and they are all older and have been working at the facility for over 20 years can make it extremely catty. I have been bullied on so many occasions now that I have lost count” [Participant from ESB country]*

*“Sometimes, I, as a foreign worker, feel like native workers treated me as if I do not know much about my task. I have to do extremely hard work compared to natives to prove myself that I am good at it. I can see clear favouritism in some cases.” [Participant from NESB country]*

*“No matter how hard we work but still we suffer from racial discrimination by some of the residents and staffs.” [Participant from NESB country]*

#### Appreciative Towards Their Job

Despite the challenging conditions of residents or the situations they faced, both groups of participants acknowledged that the positive impacts of their care on residents’ lives made a difference to their confidence to care.

*“Working in aged care makes you feel like you are making someone’s life better just by doing the smallest task for them.” [Participant from ESB country]*

Participants from NESB countries appreciated the familial connection they felt with residents that reminded them of their older relatives and kept them going in the job:

*“When I am working in the aged care, I feel like I am with my grandparents even though I am far from my family and grandparents.” [Participant from NESB country]*

#### COVID-19 Pandemic

While NESB participants did not comment on the impact of the COVID-19 pandemic on caring self-efficacy, a few participants from ESB countries reported that the COVID-19 outbreak made their work even more stressful and that the isolation, confusion, and frustrations experienced by all involved contributed to lower mental health:

*“[I’ve] noticed that [my] mental health has declined, making work more challenging and overwhelming.” [Participant from ESB country]*

Participants felt that their workload had increased, and they could not support older residents in the way they would have liked:

*“The aged people are like your family, and it’s not fair the way they are neglected [in] this time of covid. Residents just need a hug or a conversation, but [there is] no time to provide for their needs.” [Participant from ESB country]*

On the other hand, PCAs reported that being the only people the residents would see during the lockdown helped them develop personal bonds with residents, enhancing their confidence to look after them:

*“My empathy levels have increased, and [my] love for the job has increased ten-fold.” [Participant from ESB country]*

## Discussion

This study is the first to separately assess and compare the caring self-efficacy of PCAs from English-speaking and non-English-speaking countries and understand the associated factors from their perspectives. In bivariate analysis, the caring self-efficacy of PCAs born in ESB countries was significantly higher than those born in NESB countries, supporting our hypothesis. However, when country of birth (ESB vs. NESB) was adjusted with potential sociodemographic and work-related covariates, we found that the caring self-efficacy of PCAs was influenced by their age, everyday discrimination experiences, and whether they spoke English at home or not rather than where they were born. The higher mean score on caring self-efficacy reported by the ESB PCAs could have been a result of their older age and higher use of English at home. This study also found that PCAs working full-time and those with higher job satisfaction had higher confidence in their ability to care than those working part-time.

Unlike previous research (Charlesworth & Isherwood, 2021; Stevens et al., 2012), this study did not observe significant discrimination in the employment status or working hours of PCAs born in NESB countries compared to their ESB colleagues. In Australia, using 2016 Aged Care Workforce Census data, Charlesworth and Isherwood (2021) observed small group differences in both casual employment status and whether PCAs were fulltime or part-time employees, and found that being born in an NESB country predicted both casualisation and under-employment. They interpreted this as evidence of discrimination against employees from NESB backgrounds. Similar small differences in casual status and working hours were observed in the current study but the relatively low sample size meant that they were not statistically significant. However, like the Stevens et al. (2012) study, the current study identified possible discrimination in qualitative accounts of workers’ experiences.

Residents’ care preferences are often influenced by their cultural background and lifestyles before entering residential aged care (Larke et al., 2021; Walker & Paliadelis, 2016). PCAs from NESB countries may not understand or know much about older residents' cultures, traditions, or spiritual values (Chen et al., 2020). When exacerbated by limited English proficiency, PCAs from migrant backgrounds may struggle to communicate and explore these issues confidently (Gao et al., 2015), resulting in difficulty in building relationships with older residents and relatively low caring self-efficacy. On the other hand, the greater chance of a match between the language and cultural backgrounds of PCAs and those of residents when PCAs were from ESB countries may help explain their higher caring self-efficacy.

This study showed that confidence in providing quality care increased with age. This finding is not surprising, given that older PCAs are likely to have developed more coping strategies and completed more training to help them deal with their work challenges. It could also be that the older PCAs working in aged care for longer have more emotional resilience and can stay positive irrespective of the work stressors (Duffy et al., 2009). It is also possible that this result is due to survivorship bias – PCAs who find the work difficult and unrewarding do not remain long in aged care (Dickinson & Painter, 2009).

As expected, caring self-efficacy was strongly associated with the experience of everyday discrimination. However, PCAs from NESB countries were less likely to report discrimination experiences than PCAs from ESB countries, which was a surprising finding. A Canadian study reported that native-born people often report everyday discrimination due to their gender, age, weight, or sexual orientation, while race is the most common reason for experiencing discrimination among immigrants (Vang & Chang, 2019). The current study’s qualitative component identified similar explanations, where a participant from an ESB country reported her relative youth as a factor in her experience of bullying. In contrast, participants from NESB countries cited supervisors’ bias and residents’ discriminatory behaviours because of their race or migrant background. Although no previous studies have looked into whether the experience of everyday discrimination reduces caring self-efficacy, people who experience discrimination usually have low general self-efficacy (Temple et al., 2020).

The other factors that negatively affected the caring self-efficacy in both groups, as illustrated by the qualitative findings of this study, were lack of resources, lack of organisational support and recognition, and residents’ challenging behaviours, while the connectedness with residents promoted caring self-efficacy. Previous studies have reported similar findings (Australian Productivity Commission, 2011; Shrestha et al., 2021). A positive workplace culture that promotes autonomy, trust, and support at the workplace improves aged care workers' self-efficacy as carers, whereas insufficient staffing increases job stress (Shrestha et al., 2021). A previous Australian study similarly emphasised that inadequate staffing, lack of recognition at work, and unsupportive management increase aged care workers’ stress, reducing their confidence to carry out the job and increasing their intention to leave the sector (Xiao et al., 2021). On the other hand, high caring self-efficacy in care workers is associated with confidence in dealing with challenging behaviours, positive relationships with residents, and recognition of the value of their work for residents (Shrestha et al., 2021).

In the case of the COVID-19 pandemic, very few participants from either group felt that the pandemic had influenced their caring self-efficacy. Among them, only ESB participants explained how the pandemic affected their caring self-efficacy in responses to open-ended question. The impacts of the COVID-19 pandemic, such as declining mental health and increased workload, as expressed by ESB participants of this study, aggravated the PCAs’ doubt in their ability to provide quality care. While stress due to workload or inadequate organisational resources was already evident before the pandemic (Karantzas et al., 2012), these situations worsened, affecting staff members’ mental health (Krzyzaniak et al., 2021; White et al., 2021). A Belgian study conducted in 2021 found that staff felt unprepared for the situation they faced during the pandemic and expressed an urgent need for support from management to deal with the stress, both for themselves and the older residents (Kaelen et al., 2021). Staff were continuously challenged by conflicting information and protocols and were caught in professional and ethical dilemmas, such as having to choose between infection prevention and social interaction to support residents’ well-being (Kaelen et al., 2021). Care staff from NESB countries could have been further affected by their inability to visit their country of origin because of the pandemic-inflicted travel restrictions (Brydon et al., 2022). However, none of the NESB participants of this study spontaneously explained the pandemic’s impact on their caring self-efficacy.

Other study findings should be taken into consideration when assessing the validity of the study’s results. The academic qualification of PCAs from NESB countries was considerably higher than that of PCAs from ESB countries. Charlesworth and Isherwood (2021) also found that NESB PCAs were more likely to have university degree than ESB PCAs. The current study also found that many PCAs from NESB countries did not intend to continue working in aged care, which is supported by the literature. For instance, a previous study found that the turnover rate of NESB PCAs was 33% higher than that of Australian-born PCAs (Halvorsen et al., 2015). More PCAs from South and East Asia than from other regions are seeking new employment or planning to pursue postgraduate degrees (Isherwood & King, 2017). The higher qualification and turnover of NESB PCAs may be because many of them enter Australia as international students on a temporary visa and seek aged care work as a temporary employment opportunity rather than a career (Australian Government Department of Health, 2020; Howe, 2022; Morrison-Dayan, 2019).

This research concluded that the primary drivers of caring self-efficacy were English as a first language, older age, and less experience of everyday discrimination, rather than whether PCAs were born in ESB or NESB countries. Similarly, qualitative results reinforced the view that any form of discrimination and the lack of organisational support via resources, training, and supervision reduces PCAs’ confidence to do their job effectively, regardless of their country of birth.

### Implications

This study has emphasized the need for organisational resources and training opportunities for younger PCAs from both native-born and migrant groups. In addition, evidence-based training resources, such as The Little Things (Meaningful Ageing Australia, 2022), designed for use with PCAs whose first language is not English, can be used to enhance PCAs’ communication skills with residents.

This study and previous evidence highlight the fact that PCAs’ roles in residential aged care settings are mostly undervalued (Aged Care Workforce Strategy Taskforce, 2018). Aged care providers can help PCAs feel appreciated and valued by ensuring job security and flexibility in work scheduling, as well as providing financial incentives like paid leave or bonuses, and other informal incentives at work (Montague et al., 2015). Studies have shown that such employee benefits can have a large impact on employees’ motivation to work and their productivity in the job (Hong et al., 1995; Montague et al., 2015).

A comprehensive approach is recommended to combat intentional bullying of and discrimination against PCAs in RACFs, especially based on culture or country of birth. This approach should include evidence-based intervention at several levels, including anti-bullying and anti-discrimination policies to prevent unacceptable behaviours, maximise equality and opportunity for all staff, and facilitate a fair working environment (Ferris et al., 2021; Gillen et al., 2017). When necessary, in-service training should be provided to remind staff what constitutes bullying and discrimination and how to avoid those behaviours. PCAs should be able to report instances of any form of intolerant behaviours anonymously to the appropriate authority.

Similarly, age care providers must support PCAs’ mental health and their resilience to cope with negative effects of stress at work during or after the pandemic by implementing evidence-based interventions. Previous studies suggest that interventions that are flexible, culturally appropriate, adaptable, and tailored to meet local needs are key to successful implementation (Pollock et al., 2020).

### Study Limitations

There are a few possible limitations to this study. Firstly, this study was subject to sample selection bias because of the online mode of data collection. Online surveys are mainly responded to by those who are electronic-literate, have internet access, and are interested in the subject (Andrade, 2020; Bethlehem, 2010). On the other hand, the online survey reduced the risk of social desirability bias in responses because the impersonal nature of interaction in self-administered modes reduces the respondent’s sense of disclosing their answer to a third party, subsequently lowering their tendency toward socially desirable reporting (Berzelak & Vehovar, 2018; Kreuter et al., 2009). Secondly, there was a higher risk of acquiescence bias among the NESB than ESB respondents, given the relatively low levels of individualism in the cultures of their countries of birth (Johnson et al., 2005; Ross & Mirowsky, 1984). Thirdly, while multivariate modelling helped address the bias associated with the convenience sampling of participants, not all potential factors, such as supervisor’s support, burnout, organisational pressure, or training opportunities, were measured and included in the model.

Finally, most of the PCAs from NESB countries in this study came from Asian countries. NESB PCAs in the Australian aged care workforce also mainly come from Asian countries (Negin et al., 2016). However, the study results should be generalised cautiously to other groups of NESB PCAs in Australia. In addition, this study was limited to residential aged care facilities, so findings cannot be generalised to home-based aged care services.

## Conclusion

This study has added to the existing literature by recognising and exploring the caring self-efficacy of NESB PCAs, who represent almost one-third of the total PCAs working in residential aged care settings in Australia. The caring self-efficacy of PCAs was influenced by whether they primarily spoke English at home or not rather than where they were born. PCAs who were older in age and whose first spoken language was English had significantly higher caring self-efficacy than younger PCAs and those who used languages other than English at home. The experience of everyday discrimination was associated with lower caring self-efficacy. Limited or no access to organisational resources and experience of bullying and discrimination was perceived to reduce the caring self-efficacy of PCAs from both English-speaking and non-English-speaking backgrounds.

While this study supports the prospects of using migration to compensate for the labour shortage in the residential aged care sector, it also may inform aged care providers and managers about how to intervene to increase the caring self-efficacy of PCAs regardless of their country of birth. It is recommended that aged care providers develop evidence-based anti-bullying and anti-discrimination policies and practices to mitigate unacceptable behaviours based on age, language, culture, or country of birth in RACFs. Also, PCAs who are younger or who come from countries where English is not the main language should be given priority for professional development opportunities. This will help improve their caring self-efficacy and, in turn, the quality of care they provide for older residents.

## Supplemental Material

Supplemental Material - Caring Self-Efficacy of Personal Care Attendants From English-Speaking and Non-English-Speaking Countries Working in Australian Residential Aged Care Settings Click here for additional data file. Supplemental Material for Caring Self-Efficacy of Personal Care Attendants From English-Speaking and Non-English-Speaking Countries Working in Australian Residential Aged Care Settings by Sumina Shrestha, Yvonne Wells, Christine While, and Muhammad Aziz Rahman in Journal of Aging and Health.
