# Supplementary material for: Caring Self-Efficacy of Personal Care Attendants From English-Speaking and Non-English-Speaking Countries Working in Australian Residential Aged Care Settings
Source: J Aging Health. 2023 Jun 14;36(3-4):207–19. doi: 10.1177/08982643231183466 (PMC10832313; doi:10.1177/08982643231183466)
Supplement: Supplemental Material - Caring Self-Efficacy of Personal Care Attendants From English-Speaking and Non-English-Speaking Countries Working in Australian Residential Aged Care Settings [file sj-pdf-1-jah-10.1177_08982643231183466.pdf]

Supplementary Table

Mean scores of caring self-efficacy, confidence to care and no doubts and concerns based on sociodemographic and work-related characteristics

**Variables**	**Category**	**Mean score** ± SD		
		**Caring Efficacy**	**Confidence to care**	**No Doubts & Concerns**
Gender	Female	115.1 ± 12.8	54.8 ± 5.8	60.2 ± 8.8
	Male	114.8 ± 8.0	52.6 ± 3.4	62.2 ± 6.4
Age	18 - 32 years	110.4 ± 14.1	52.8 ± 6.4	57.5± 9.9
	33 – 47 years	115.7 ± 11.3	54.9 ± 5.5	60.8 ± 7.6
	48 to 67 years	119.5 ± 10.1	56.6 ± 4.5	62.9 ± 7.5
Marital status	Never Married	115.4 ± 12.2	55.0 ± 5.3	60.4 ± 8.7
	Ever Married	114.9 ± 12.7	54.6 ± 6.0	60.3 ± 8.8
Educational level	Diploma or degree	112.2 ± 13.6	53.5 ± 6.4	58.7 ± 9.2
	Certificate III/ IV only	117.1 ± 11.3	55.6 ± 5.1	61.5 ± 8.2
Country of birth	English speaking countries	117.4 ± 11.1	55.9 ± 4.8	61.6 ± 8.0
	Non-English-speaking countries	109.8 ± 13.8	52.2 ± 6.8	57.7 ± 9.6
Language spoken at home	English	117.3 ± 11.3	55.8 ± 5.0	61.6 ± 8.1
	Others	107.6 ± 13.4	51.3 ± 6.7	56.3 ± 9.4
Feeling Australian	Very/Somewhat Australian	115.9 ± 12.1	55.2 ± 5.4	60.7 ± 8.5
	Not very/not at all Australian	109.5 ± 13.7	51.5 ± 6.8	58.1 ± 9.7
Everyday discrimination	Low experience	118.4 ± 12.5	55.7 ± 5.6	62.7 ± 8.7
	Middle	114.1 ± 10.2	54.5 ± 5.1	59.6 ± 7.6
	High experience	112.6 ± 13.9	53.9 ± 6.4	58.6 ± 9.2
**Work-related variables**				
Informal care experience	Yes	115.1 ± 12.6	54.9 ± 5.2	60.3 ± 8.9
	No	114.9 ± 12.5	54.3 ± 6.4	60.6 ± 8.4
Years of experience	Lowest (≤ 3 years)	111.6 ± 13.8	53.2 ± 6.3	58.4 ± 9.7
	Middle (4-8 years)	117.6 ± 10.9	55.9 ± 5.4	61.8 ± 7.6
	Highest (≥ 9 years)	116.6 ± 11.6	55.4 ± 5.0	61.2 ± 8.1
Intention to remain in aged care job	Yes	116.2 ± 12.4	55.1 ± 5.6	61.1 ± 8.7
	No	112.5 ± 12.5	53.9 ± 6.0	58.6 ± 8.6
Employment status	Permanent	115.6 ± 12.2	54.9 ± 5.8	60.7 ± 8.4
	Contract/Casual	113.0 ± 13.4	54.0 ± 5.5	59.0 ± 9.8
Working hours	Full-time	118.0 ± 13.3	56.2 ± 5.4	61.8 ± 9.4
	Part-time	114.2 ± 12.2	54.3 ± 5.8	59.9 ± 8.5
Type of facility	Private	115.0 ± 11.7	54.7 ± 5.2	60.3 ± 8.5
	Others	115.1 ± 13.3	54.7 ± 6.2	60.4 ± 9.0
Job Satisfaction	Satisfied	115.5 ± 12.9	54.8 ± 5.8	60.7 ± 8.9
	Not satisfied	113.5 ± 11.1	54.5 ± 5.5	59.0 ± 8.1
Perceived quality of training	Excellent/Good	115.3 ± 12.4	54.8 ± 5.8	60.6 ± 8.6
	Fair/Poor	113.5 ± 13.4	54.4 ± 5.7	59.2 ± 9.3
Influence of COVID pandemic on the CES	Yes	113.5 ± 12.3	55.0 ± 4.9	58.6 ± 9.2
	No	115.2 ± 12.6	54.7 ± 5.9	60.5 ± 8.7
